# Determining six-month prognosis among people with dementia living in care homes: a systematic review of prognostic tools

**DOI:** 10.1093/ageing/afag087

**Published:** 2026-04-10

**Authors:** Emily West, Laura Mulligan, Prabin Paudyal, Terence J Quinn, Jennifer Kirsty Burton

**Affiliations:** Academic Geriatric Medicine, School of Cardiovascular and Metabolic Health, University of Glasgow, Glasgow, Scotland, UK; Department of Medicine for the Elderly, Glasgow Royal Infirmary, Glasgow, Scotland, UK; Department of Medicine for the Elderly, Glasgow Royal Infirmary, Glasgow, Scotland, UK; School of Medicine, Dentistry & Nursing, University of Glasgow, Glasgow, UK; Medicine for Older Adults, University Hospital Hairmyres, East Kilbride, UK; Academic Geriatric Medicine, School of Cardiovascular and Metabolic Health, University of Glasgow, Glasgow, Scotland, UK; Department of Medicine for the Elderly, Glasgow Royal Infirmary, Glasgow, Scotland, UK; Academic Geriatric Medicine, School of Cardiovascular and Metabolic Health, University of Glasgow, Glasgow, Scotland, UK; Medicine for Older Adults, University Hospital Hairmyres, East Kilbride, UK

**Keywords:** dementia, long-term care, prognosis, palliative and end of life care, care home, systematic review, older people

## Abstract

**Background:**

Dementia is a leading cause of death among residents in care home settings internationally. Proactive identification of those approaching end-of-life can support future care planning, respecting preferences. We aimed to synthesise existing literature on prognostic tools designed to predict 6-month mortality in individuals with dementia residing in care homes including their content and prognostic performance.

**Methods:**

A systematic review was undertaken, searching Embase, CINAHL, PsycINFO and MEDLINE in December 2024. Studies evaluating prognostic tools for predicting 6-month mortality in individuals with dementia living in care homes were included. Risk of bias was assessed using the PROBAST tool. Data were extracted and narratively synthesised.

**Results:**

Thirteen studies involving 503 501 participants were included from a total of 5438 records screened. Eleven prognostic tools were identified. Only three studies were assessed at low risk of bias. Predictive factors per tool ranged from 4 to 12. Age, change in cognitive function, functional decline/dependency and concerns around nutritional intake/weight were the commonest domains of predictors across included tools. Reporting around the discriminative performance of tools was limited and only two tools (Mortality Risk Index Score and Advanced Dementia Prognostic Tool had acceptable discrimination (area under receiver operating curve >0.70), which was not replicated in validation studies.

**Conclusions:**

While identified predictors have clinical congruence, there is significant variability in how these are assessed and operationalised and in the application of prognostic tools. Specific prediction of mortality remains challenging and would benefit from further research to adapt and validate dynamic prognostic tools for use in practice.

## Key Points

- Our review identified significant heterogeneity in prognostic tools for people living with dementia in care homes
- Two tools using data from the minimum data set were identified with acceptable discrimination, which has not been externally replicated
- There has been little new research in dementia prognostication over the past decade
- Further research is needed to adapt and externally validate a dynamic prognostic tool which could be used in this population

## Introduction

Dementia is a progressive neurodegenerative disease affecting ~57 million people worldwide, with estimates of a growing global population of people living the condition [1]. As the disease advances, individuals often require additional support with activities of daily living (ADL), including continence, personal care and management of neuropsychiatric symptoms [2]. The majority of people living with dementia are also living with multiple long-term conditions and this is associated with poorer outcomes [3].

While there is a lack of consensus around the optimal time for moving-in to a care home setting for individuals living with dementia, [4] the condition itself is strongly associated with the need for care placement [5, 6]. A recent systematic review estimated that individuals diagnosed with dementia can spend up to a third of their remaining life expectancy in a nursing home setting [7]. Thus, dementia is common in people living in care home settings [8, 9]. As the number of people living with dementia grows, so the numbers dying with it are estimated to increase significantly, [10, 11] commonly in care home settings [12]. To-date, dementia of all causes accounts for over one-third of all deaths among care home residents in Scotland [13].

It is well-documented that those with dementia experience barriers to accessing high quality palliative care. Reasons include a widespread failure to recognise dementia as a terminal illness, [14, 15] fragmented access to healthcare services [16, 17] and lack of access to specialist palliative care support [18]. Timely identification of individuals in the last months of life is essential to avoid unnecessary hospital admissions and medical interventions that may be burdensome [19, 20]. Recent data from London supports that where palliative care needs are prospectively identified, there is a lower risk of multiple unscheduled hospitalisations and greater engagement of primary and community care support [21]. However, prognostication in dementia remains an area of challenge in clinical practice due to the clinical trajectory, impact of other long-term conditions and organisation of palliative care services which remain focused around other conditions [15].

Prognostication among adults living with chronic disease is an area of active interest for clinicians and researchers [22, 23]. A 2012 systematic review considered 6-month prognosis in people living with advanced dementia across all settings, concluding there was a lack of prognostic concordance across the literature [24]. A 2020 scoping review of prognostic variables in dementia identified 239 factors across six thematic areas which had been examined in the published literature and variation in predictive factors based on the setting of care [25]. This review grouped hospices and care home settings together in their analysis of individual factors [25]. Despite most dementia-related deaths taking place in care homes, no previous review has evaluated the performance of 6-month prognostic tools for dementia in long-term care settings.

The aim of this systematic review was to establish what tools exist to identify 6-month prognosis among people living with dementia in care homes and to assess their content and performance.

## Methods

This review was reported following Preferred Reporting of Items in Systematic Reviews and Meta-Analyses (PRISMA) guidance [26]. The protocol was registered on PROSPERO in 2024 (CRD: 42024623940: https://www.crd.york.ac.uk/PROSPERO/view/CRD42024623940).

### Eligibility criteria

Studies reporting on prognostic models were included. These were based on observational cohort studies both prospective and retrospective in design.

We placed no restrictions on date or language of publication. If abstracts were identified, we searched for subsequent full-text publications using author names and abstract titles. If no full text publication was identified, the abstract was included, with limited data extraction and without risk of bias assessment.

#### Population

People living in care homes who have a diagnosis of dementia of any subtype. We included dementia however this was defined, by study authors, but did not include those diagnosed with mild cognitive impairment. Where studies included people living with and without dementia, we assessed eligibility for inclusion in this review based on whether results data was presented about those living with dementia separately.

#### Outcomes

Identification of prognostic models/tools for 6-month mortality among people living with dementia, including a summary of their accuracy and performance. The review was interested in the performance of the model/tool including the number and range of predictors, discrimination, calibration and any other relevant statistical summaries.

Other pre-specified outcomes of interest were the applicability of models to specific subgroups: based on dementia sub-type and the applicability of evidence for individuals with other comorbidities alongside dementia—e.g. cancer, vascular disease and other neurodegenerative disease.

#### Timing

Six month prognosis has been previously identified as clinically significant due to the association with funding for hospice care in some jurisdictions [27, 28] and is a meaningful period for people and their families. We also explored the timing of when a prognostic model was applied for a person living in a care home setting with dementia. We were interested in identifying tools which could be used in practice to identify a resident was approaching their last months of life.

#### Setting

Our study focused on people living in care homes. Recognising heterogeneity in the terminology, this was inclusive of synonymous long term care settings internationally providing 24-hour residential care and support for adults. [29, 30] Where studies included a wider population of people living with dementia, beyond care homes, we included data from the study where results data relating to the care home population was available separately.

### Information sources

We searched MEDLINE (Ovid); EMBASE (Ovid), CINAHL (EBSCOhost) and PsycInfo (EBSCOhost) from 1980 to December 2024. The search was developed involving three concepts—people living with dementia (‘population’), prognostic tools for mortality (‘concept’) in care home settings (‘context’). The full strategy is reported in Appendix S1, it was designed drawing on other published review search strategies [6, 31–34]. We checked reference lists of identified systematic or scoping reviews to identify any additional studies.

### Study selection and data collection

We used Covidence software [35] for deduplication and study selection. Title/abstract screening was undertaken by independent pairs of reviewers (EW, LM, PP and JKB). Conflicts were resolved by team discussion, involving senior authors (JKB and LM). Full text screening followed the same approach.

### Data extraction and risk of bias assessment

We developed an electronic data extraction form and piloted this on a sample of four papers, making adaptations for consistency and completeness. Independent data extraction by two authors (EW and LM) was undertaken for all full-text papers, and a single author (JKB) undertook data extraction for those where only the abstract data was available.

Data were extracted on: year of publication; country/countries; study design; number of care homes; description of care home setting; number of participants; age; sex; race/ethnicity; diagnostic criteria for dementia; dementia subtype; severity score to define advanced dementia; other comorbidities; causes of death; follow-up duration; 6-month survival; prediction model; data source; statistical modelling; number of predictors; list of predictors; timing of prediction; discrimination; calibration; other statistics reported and any comparison to other scores or tools.

A narrative synthesis of results is presented due to the significant heterogeneity between included studies. We grouped predictors into common domains to look at their distribution across the included studies. We found two types of predictive tools: tools specifically designed for predicting mortality in people living with dementia in care homes and a second group of tools designed for broader use in predicting mortality outcomes applied to a care home population. We reported where studies had evaluated discrimination and calibration and used recognised cut-off points to describe the level of discrimination based on area under receiver-operating-characteristics curves [23].

Risk of bias was evaluated by a single author (JKB) using the PROBAST tool which is specific for use in prediction model studies [36]. Four assessments of risk of bias are made individually (across the domains of participants, predictors, outcome and analysis) then three assessments of applicability are made about the included participants, predictors and outcomes. These are then considered together to present an overall judgement for each study. We used the risk of bias Excel template developed by Fernandez-Felix *et al.* to enable generation of a graphical summary of findings [37]. Risk of bias assessment was undertaken for full-text studies only, due to the lack of detail available from conference abstracts alone.

## Results

**Figure 1:**
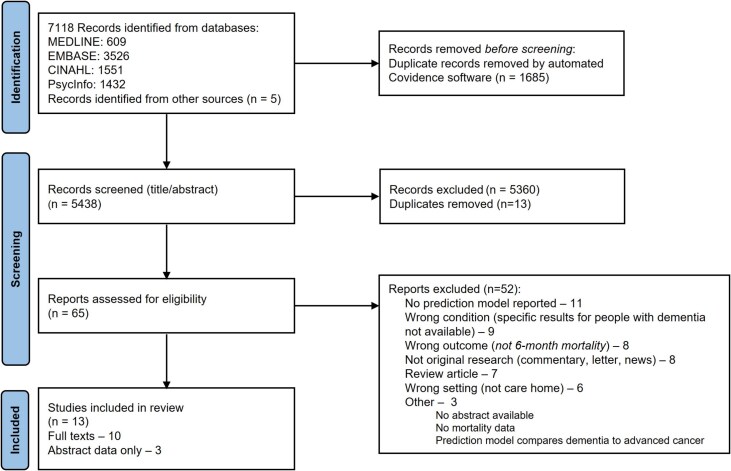
PRISMA diagram [46].

We screened 5438 records by title and abstract after automated de-duplication. Five additional studies were identified from the reference lists of similar review articles. We assessed 65 studies in full and included 13 publications, three in abstract form only. We excluded 52 studies with main reasons for exclusion including *n* = 11 no prediction model reported; *n =* 9 wrong condition (results not available for people with dementia separately); *n =* 8 wrong outcome (not 6-month mortality); *n =* 8 not original research. All reasons for exclusion are reported in Figure 1. Of the eight studies which looked at prediction of a different mortality time-point, these included 14 days, [38] 30 days, [39] 1 year, [40, 41] 19 months, [42] 2 years [43, 44] and 5 years [45].

### Included study characteristics

The review includes ten original articles [47–56] and three abstracts [57–59], included study characteristics are summarised in Table 1. Included studies were mainly of cohort design, with six being retrospective [52, 53, 55, 57–59]. Nine studies were conducted in North America, [48–54, 56, 58] with one in both North America and the Netherlands [55]. All were situated in high-income countries.

**Table 1:** Included study characteristics: published papers and conference abstracts.

Study ID	Country	Sample size	Number of care homes	Age in years (mean +/− SD unless stated)	Female sex (%)	How dementia diagnosed and included subtype(s)	Severity inclusion criteria	6-month survival (%)	Risk of bias and applicability
Published papers									
Esteban-Burgos 2023 [47]	Spain	149 146	8	84.7 +/− 9.3	70.6	Not reported	Not reported	74.8	R: high A: high
Hicks 2010 [48]	USA	123	3	81.5 +/− 7.1	55.3	Diagnosis of dementia in medical record All subtypes: 57.7% Alzheimer’s, 42.3% others^a^	Met hospice criteria for dementia or were receiving hospice or palliative care	68.0	R: high A: high
Hirdes 2014 [49]	Canada	118 429	Not reported	Age distribution: 2.6% <65, 7.4% 65–74, 32.7% 75–84, 57.4% >84	68.3	Resident Assessment Instrument (RAI 2.0) diagnosis of Alzheimer’s and related dementias	Not reported	Not reported	R: high A: high
Marsh 2000 [50]	USA	112	5	82.0 +/− 7.7	75.0	Diagnosis of Alzheimer’s dementia	GDS >6	80.4	R: high A: high
McCusker 2014 [51]	Canada	180	7	>65	56.0	Diagnosed by a physician on resident chart Subtypes not reported	Not reported	89.0	R: high A: high
Mitchell 2004 [52]	USA	11 430 (6799 derivation; 4631 validation)	1082 (642 derivation; 440 validation)	Median 83	66.8	Diagnosis of Alzheimer’s and other dementias in MDS data	Cognitive performance score 5–6	68.96% (71.7% derivation; 64.9% validation)	R: low A: low
Mitchell 2010(a) [53]	USA	22 405	Not reported	84.5 +/− 7.5	77.0	Diagnosis of dementia in MDS data	Cognitive performance score 5–6 (from MDS)	Not reported	R: low A: low
Mitchell 2010(b) [54]	USA	606	21	85.0 +/− 7.0	81.9	Dementia diagnosed in medical record	Cognitive performance score 5–6 (assessed prospectively)	81.7	R: high A: high
Van der Steen 2007 [55]	Netherlands and USA	557 (288 Dutch; 269 USA)	41 (6 Dutch; 35 USA)	Proportion > 83 Dutch 56.6%; USA 62.8%	74.7	Diagnosis of Alzheimer’s and other dementias in MDS data	Cognitive performance score 5–6	69.7 (Dutch 75.7; USA 63.2)	R: low A: low
Volicer 1993 [56]	USA	139 (development 68; testing 71)	3	67.5 +/− 0.8	3	Neurologist clinical evaluation of Alzheimer’s dementia using DSM 3 criteria	Not reported	66.2	R: high A: high
Conference abstracts									
Almeida 2012 [57]	Not reported	24	1	Not reported	Not reported	Dementia as cause of death Subtype not specified	Not reported	Not reported	Not assessed
Voldberding 2013 [58]	USA	236	1	Not reported	Not reported	Not reported	Not reported	36.9	Not assessed
Wong 2018 [59]	Hong Kong	114	Not reported	Not reported	Not reported	Clinical management system data Subtype not specified	Not reported	50.0	Not assessed

Abbreviations: DSM—Diagnostic and Statistical Manual; SD—standard deviation.

R—risk of bias; A—applicability (full reporting of risk of bias assessment included in Appendix S2 and Appendix S3).

^a^Vascular dementia, mixed dementia, Lewy body dementia, frontotemporal dementia, dementia type not specified, dementia due to HIV, dementia due to Parkinson disease.

Eight studies were undertaken in nursing homes, four were in settings described as long-term care facilities, institutions or homes [49–51, 56] and one in a dementia special care centre [58]. Across ten studies, residents in 1172 care homes were represented, three studies did not report the total number of care homes they included [49, 53, 59].

### Included participant characteristics

The total review population includes 503 501 people. Individuals study sample sizes varied from 24 to 222 405 participants (median = 236, mean = 38 730). Average sample size is significantly skewed by three large studies of >100 000 participants [47, 49, 52].

#### Demographics

The average (typically reported as a mean) age of participants was between 67.5 and 85 years. Three studies did not report average age, with one providing distribution in age bands, [49] one specifying all had to be >65 years [51] and one reporting the proportion aged >83 years [55]. Women accounted for the largest proportion of participants in all studies apart from one, where they accounted for just 3% [56]. The authors of the latter study did not provide further explanation on their imbalanced population. Only six studies reported on participant ethnicity and those from white ethnic groups accounted for the majority of all six studies [48, 50, 52–54, 56].

#### Dementia

Dementia severity was assessed in six studies. This included meeting hospice criteria for dementia, [48] a Global Deterioration Scale (GDS) greater than six [50] and four studies who defined severity based on Cognitive Performance Score of five or six [52–55].

Two studies included only patients with Alzheimer’s type dementia, [50, 56] five studies enrolled patients with Alzheimer’s and other dementia types [48, 49, 52–54]. The remaining six studies did not define the subtypes of dementia included in their study. Only one study presented quantitative data on the proportion of their sample living with Alzheimer’s dementia versus other dementia subtypes [48].

#### Comorbidity

Three studies used formal tools to quantify comorbidity—one used the weighted index of comorbidity [50] and two others used the Charlson Index [51, 59]. Two studies did not measure comorbidity among included participants [57, 58]. Of the remaining studies, approaches to comorbidity reporting included five studies which reported the prevalence of a range of conditions, [47, 48, 52, 53, 56] while three other studies took a specific focus on conditions of interest including lower respiratory tract infection, [55] congestive heart failure, [54] or eleven neurological conditions [49].

#### Timing of prediction tool application

Five studies used time of enrolment into a research project as the timing of prognostication [47, 48, 50, 54, 59]. Three of these focused on enrolling those thought to be approaching end of life, based on meeting hospice criteria [47, 48] or clinician judgement [59]. Three studies applied mortality prediction at the time of nursing home admission [51–53]. Two studies used retrospective review of cases who died and the timing of tool application is unclear from the abstract data available [57, 58]. One study evaluated prognostic factors for survival following an episode of fever [56]. One study used the most recent assessment data recorded using the Resident Assessment Instrument, the study authors anticipated this assessment would have been made within the last 3 months [49]. The final study applied different approaches to tool application, with one cohort meeting a clinical definition of advanced dementia and the other in residents with advanced dementia who had experienced lower respiratory tract infection [55].

#### Follow-up and mortality

Participants were followed up for a duration between six and 71 months (average 23.6 months).

None of the studies reported causes of death. Survival at 6 months ranged from 36.9%–89.0% (average 68.5%). Three studies did not report 6-month survival [49, 53, 57].

### Risk of bias within studies

Three studies were considered overall to have low risk of bias and low concerns around applicability [52, 53, 55]. Whereas the remaining seven were considered at overall high risk of bias and high concern around applicability [47–51, 54, 56]. These are summarised in Appendices S2 and S3.

Issues in the participant domain largely related to retrospective study designs and selection bias around the involvement of people living with dementia in care home settings. This was commonly due to recruitment concerns where individual/proxy consent to participate was needed. Predictor assessment was at low risk of bias across all studies, largely as predictors were evaluated using consistent national datasets or standardised researcher assessments. Outcome assessment methods varied, but this domain was also considered at low risk of bias across all studies due to the nature of the outcome being assessed as binary and concrete. The most significant concerns were around the analysis approach adopted and included selection of predictors based on univariate performance, the lack of evaluation of model performance including accounting for overfitting and optimism in performance.

### Prognostic tools

Six studies report on the development of a mortality risk prediction tool [48, 50–53, 56]. Two studies report on validations of existing mortality risk prediction tools [49, 55] and two studies compare the performance of existing mortality risk prediction tools in the care home population [47, 54]. Of the three publications in abstract version only, two examined the performance of existing tools [57, 58] and one described developing a new tool [59].

**Figure 2:**
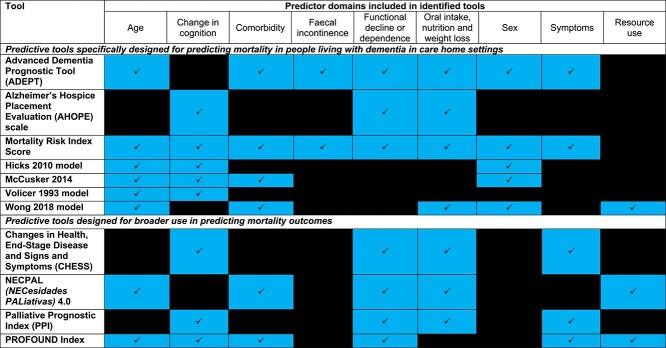
Mapping predictor variable domains used in included tools.

The prognostic tools examined in the included studies, and the predictor variables included are summarised in Table 2. The degree of overlap between tools in core domains are presented in Figure 2. This is split to display domains included in the seven tools specifically designed for predicting mortality in people living with dementia in care home settings, compared to the four tools with broader use. No tool included all domains. Age and change in cognitive function were the most common domains included in eight tools followed by functional decline or dependency and oral intake/nutrition/weight loss, both examined in seven tools.

**Table 2:** Summary of prognostic tools and their included predictors.

Study ID	Prognostic tool evaluated	Data source to derive predictors	Number of predictors	Predictors included in the tool	
Almeida 2012 [57] Mitchell 2004 [52] van der Steen 2007 [55] Volberding 2013 [58]	Mortality risk index score	MDS	12	ADL scale Age > 83 years Bedbound Bowel incontinence Cancer Congestive heart failure	Male sex Not awake most of the day Shortness of breath Unstable medical condition Use of supplementary oxygen <25% of food eaten at most meals
Esteban-Burgos 2023 [47]	NECPAL 4.0	Original data collection	7	Age Disease specific criteria of severity (*For dementia global deterioration score > 6*) Functional decline	Multimorbidity Nutritional decline Palliative care needs identified by healthcare professional Use of resources
	Palliative prognostic index (PPI)	Original data collection	5	Delirium Dyspnoea at rest Oedema	Oral intake Palliative performance score
	PROFOUND Index	Original data collection	9	Age > 85 Active neoplasia Barthel index <60 Caregiver other than spouse Dementia present Delirium in last hospitalisation	Grade III–IV functional class on NYHA and/or MRC classifications Haemoglobin <100 g/l ≥4 hospitalisations in last 12 months
Hicks 2010 [48]	Model not named	Care of Nursing Home Residents with Advanced Dementia (CareAD) study	6	Age Dementia subtype Gender Pneumonia in prior 6 months	Severe Impairment Rating Scale (SIRS score) 7+ years with dementia
Hirdes 2014 [49]	Changes in Health, End-Stage Disease and Signs and Symptoms (CHESS) Scale	inteRAI assessment data from home care and continuing care	4	Clinician determined prognosis <6-months Decline in ADLs	Decline in cognition Weight loss, shortness of breath or oedema present
Marsh 2000 [50]	Alzheimer’s Hospice Placement Evaluation (AHOPE) scale	Original data collection	9	Ambulation Eye contact Food intake Fluid intake Level of consciousness	Muscle flexibility Speech Swallowing Weight history
McCusker 2014 [51]	Model not named	Original data collection	7	Age Charlson Comorbidity Index Delirium Dementia	Depression Sex Time since admission
Mitchell 2010(a) [53] Mitchell 2010(b) [54]	Advanced Dementia Prognostic Tool (ADEPT)	MDS	12	Admitted <90 days previously Age Bed bound Body mass index Bowel incontinence Congestive heart failure	Dyspnoea Insufficient intake Male sex Pressure ulcers Total functional dependence Weight loss
Volicer 1993 [56]	Model not named	Original data collection	4	Age Dementia severity (*Bedford Alzheimer Nursing Scale*)	Management strategy (*Level of care e.g. antibiotics vs palliative*) Time from admission
Wong 2018 [59]	Model not named	CMS (abbreviation not defined)	7	Age Charlson Comorbidity Index Estimated Glomerular Filtration Rate Number of hospitalisations in last 6 months	Serum albumin Sex Tube feeding

Abbreviations: interRAI—international Resident Assessment Instrument; MRC—Medical Research Council; NYHA—New York Heart Association.

Included studies poorly reported the performance of their models in terms of discrimination and calibration, with less than half providing area under curve (AUC) statistics and only four reporting on calibration (Table 3, Appendix S4). Only two models [Mortality Risk Index (also known as the MDS Risk Index) and ADEPT] had AUC values in the acceptable range (>0.70) for 6-month mortality [52, 53]. The Mortality Risk Index has been applied by others, including a joint US and Dutch validation study, in collaboration with the index authors [55]. However, the discrimination of the model in these cohorts was poor and lower than the original development study [55]. We did not identify other external validations of the ADEPT and note that the prospective validation study undertaken by the original authors demonstrated lower discrimination both when a cut-off score or continuous variable were used [54]. In the original study, using a cut-off score for ADEPT resulted in area under receiver operating curve (AUROC) 0.65 in the whole cohort and 0.59 among residents living in the home for at least 12 months [53].

**Table 3:** Reporting of discrimination and calibration across included studies.

Study ID	Prognostic tool	Discrimination of model	Evaluation of calibration
Marsh 2000 [50]	Alzheimer’s Hospice Placement Evaluation (AHOPE) scale	Not reported	Not reported
Mitchell 2010(a) [53]	Advanced Dementia Prognostic Tool (ADEPT)	AUROC 0.73	Calibration plots provided
Mitchell 2010(b) [54]	Advanced Dementia Prognostic Tool (ADEPT)	AUROC 0.67 (95% CI 0.62–0.72)	Hosmer-Lemeshow test result (P 0.69)
Hirdes 2014 [49]	Changes in Health, End-Stage Disease and Signs and Symptoms (CHESS) Scale	Not reported	Not reported
Almeida 2012 [57]	Mortality Risk Index Score	Not reported	Not reported
Mitchell 2004 [52]	Mortality Risk Index Score	Derivation: AUROC 0.74 Validation: AUROC 0.70 Sensitivity and specificity data present for each cut-off point of the score	Not reported
Van der Steen 2007 [55]	Mortality Risk Index Score	Dutch cohort AUROC 0.65 (95% CI 0.58–0.72) US cohort AUROC 0.64 (95% CI 0.58–0.71)	Assessed by comparing observed mortality over risk strata in the development cohort with observed mortality in the study cohorts
Volberding 2013 [58]	Mortality Risk Index Score	Not reported	Not reported
Esteban-Burgos 2023 [47]	NECPAL (*NECesidades PALiativas*) 4.0	AUROC 0.41	Not reported
	Palliative Prognostic Index (PPI)	AUROC 0.48	Not reported
	PROFOUND Index	AUROC 0.66	Not reported
Hicks 2010 [48]	Model not named	Not reported	Not reported
McCusker 2014 [51]	Model not named	Not reported	Not reported
Volicer 1993 [56]	Model not named	Not reported	Not reported
Wong 2018 [59]	Model not named	AUROC 0.63	Hosmer-Lemeshow test result (>0.05)

Abbreviation: CI—confidence interval.

### Subgroup analyses

Of our pre-specified outcomes of interest, no studies reported data separately based on dementia sub-type. However, one study included mixed or other dementia (versus Alzheimer’s dementia) as a variable in their predictive model, finding those with Alzheimer’s dementia and pneumonia had shorter survival than for other dementia subtypes [48].

Similarly other comorbidities were included in predictive models with congestive heart failure in two models, [52, 53] cancer in two models, [47, 52] depression in one model [51] and multimorbidity or comorbidity included in three models [47, 51, 59].

## Discussion

### Findings in context

Our systematic review identifies that there is no optimal prognostic tool to identify 6-month mortality among people living with dementia in care homes. There is significant heterogeneity across the included studies. The two tools (Mortality Risk Index and ADEPT) developed by Mitchell *et al.* in 2004 [52] and 2010 [53] respectively offer the most acceptable performance and use data from the minimum data set (MDS; structured standardised data-collection approach used internationally). However, external validation has either not replicated original model performance or not been undertaken, meaning further work is required before considering use in routine practice. This includes the use of cut-off scores and how to operationalise the tool, issues present at time of development [53]. The literature thus has not significantly advanced since the 2013 systematic review, across care settings, undertaken by Brown *et al.* [24]

The predictor variables used within tools have clinical congruence in identifying those with more advanced disease—advanced age, worsening cognition, dependency and concerns around oral intake/weight. They also align with predictor variables identified across non-communicable, non-cancer disease prediction models [22]. However, operationalising these for use in clinical practice is challenging and risks overlooking the holistic needs of an individual dying with dementia [60].

A gradually declining illness trajectory for people with dementia has been described in the literature [61]. The role of acute intercurrent illnesses, such as infections or fractures, to alter prognosis for an individual living with dementia are also increasingly recognised [62]. Pneumonia is a specific prognostic factor which has been identified previously in this population, [63] and for which a prediction model has been developed, albeit for 14-day mortality [38]. Risk prediction in this population requires to be dynamic to changing circumstances in which factors, such as infection, fever and difficulties with eating are common and associated with increased 6-month mortality [64].

### Strengths and limitations

This study benefits from structured conduct based on a pre-specified protocol and use of good practice in undertaking systematic review research. It addresses an applied clinical topic and area of uncertainty in an under-researched population. However, we have been unable to perform any quantitative synthesis based on the heterogeneity of tools identified and limited data using the same tool in different populations.

However, by focusing on those living in care homes we have excluded evidence about the population living with advanced dementia cared for elsewhere in the community and in hospital settings. This led to exclusion of a model developed in Taiwan, across the whole population living with dementia, [65] a model derived on those presenting at in acute care [66] and a deep learning algorithm developed on hospital inpatients [67]. Our focus on the care home population was intended both to consider those with complex needs and to identify if sector-specific tools exist making use of data collected in care home settings, which may not be consistently present for those elsewhere, such as the MDS or Inter-RAI (used as the source for predictors in several included studies).

We were interested in those living in care homes with dementia, but this approach excludes those living with other long-term conditions. Focusing primarily on dementia can be justified based on the prevalence of the condition, but note that some studies have looked at predicting care home mortality as a whole, based around tools such as the FRAIL-NH [68] or Mortality Risk Index [69]. These tools would also benefit from exploration in care home settings internationally.

### Implications for clinical practice and future research

For clinical audiences the message is clear that there is uncertainty in predicting mortality in this population which needs to be managed in care interactions. A recent scoping review identified that only ~40% of caregivers recognised dementia as a life-limiting condition and that their understanding of the condition is associated with the care experienced by their loved-one during the dying phase, [70] thus ensuring caregivers are informed and supported is critical. Jurisdictions providing palliative care support for those felt to be at risk of mortality demonstrate beneficial impacts on service utilisation measures [71]. However, while the components of palliative care interventions for this population have been identified, [72] delivering this in a consistent way remains a challenge and active area for research [73]. Consistent delivery of palliative care interventions may be improved if a well-performing and usable prognostic model for 6-month mortality was routinely available.

This review highlights the ongoing evidence gap around a high-performing, externally validated prognostic tool for 6-month mortality among people living with dementia in care homes. We note with interest a 2020 protocol to develop a tool for use in Australian Residential Aged Care Facilities [74]. More research is needed to determine the models which perform best in clinical practice, beyond their original settings of development. Future prognostic model studies need to be clear on how variables are defined and measured, when models are applicable and reduce bias through inclusion of more representative cohorts. For countries not using established data capture mechanisms, such as the MDS, using digital care record data collected in care home settings offers the potential to develop tools from routinely-collected data [75]. This could enable modelling of the impact of acutely changing health (as a consequence of dementia or other comorbid long term conditions) on prognosis and survival.

## Supplementary Material

Supplementary_materials_afag087

## Data Availability

In addition to the data available in the supplementary materials, the underlying data supporting this review is available on reasonable request through the corresponding author.
