# Supplementary material for: Determining six-month prognosis among people with dementia living in care homes: a systematic review of prognostic tools
Source: Age Ageing. 2026 Apr 10;55(4):afag087. doi: 10.1093/ageing/afag087 (PMC13070562; doi:10.1093/ageing/afag087)
Supplement: Supplementary_materials_afag087 [file supplementary_materials_afag087.docx]

Supplementary Materials for determining six-month prognosis among people living in care homes with dementia: a systematic review of prognostic tools

|  | Page |
| --- | --- |
| Appendix 1: Search strategy | 2 |
|  |  |
| Appendix 2: Risk of bias summary table | 3 |
|  |  |
| Appendix 3: Risk of bias and applicability summary | 4 |
|  |  |
| Appendix 4: Summary of discrimination and calibration assessment across included studies | 5 |
|  |  |

**Appendix 1: Search strategy**

| **POPULATION:** People living with dementia   - exp Dementia/ - Delirium, Dementia, Amnestic, Cognitive Disorders/ - dement*.ti,ab. - alzheimer*.ti,ab. - AD.ti,ab. - Alzheimer Disease/ - vascular dementia.mp. - Dementia, Vascular/ - Dementia, Multi-Infarct/ - (binswanger* or ((subcortic* or “sub cortic*” or arterisclerotic) adj4 (encephalopath* or leukoencephalopath*)) or cadasil*).tw. - ("lewy bod*" or DLB or LBD).tw. - PDD. tw. - ((pick* adj1 (complex or disease* or syndrome)) or (wilhemsen adj1 lynch) or ddpac or (lob* adj4 atroph*)).tw - (((frontotemporal or (fronto adj temporal) or (corticobasal or (cortico adj basal) or (frontal adj lobe))) adj4 (degenerati* or dysfunction)) or ftld or ftlds or ftd or ftds).tw. - (huntington* or ((progressive or major or juvenile or hereditary) adj4 chorea)).tw. - korsako*.tw. - (Posterior adj cortic* adj atroph*).tw. - (((creutzfeldt or ja?ob*) adj4 (disease or syndrome)) or cjd or (spongiform adj4 encephalopath*) or “corticostriatospinal degeneration” or (pseudosclerosis adj4 spastic)).tw. |
| --- |
| **CONCEPT:** Prognosis/prediction of six-month mortality   - Nursing homes/ - nursing home.mp. - (care home or care home*).mp. - Residential Facilities/ - residential.mp. - “Residential home”.mp - "long term care".mp. - Long-Term Care/ - Homes for the Aged/ - ((aged or elderly) adj2 (care or facilit* or home*)).ti,ab. - “institutional care”.mp |
| **CONTEXT:** living in care home settings   - Prognosis/ - Prognosis.mp. - Prognostication.tw - (risk* adj2 (tool* or index or indices or score* or scale* or predict*)).ti,ab. - ((prognos* or predict*) adj2 (tool* or index or indices or score* or scale*)).ti,ab. - Survival/ - Death/ - Mortality/ - “6-month mortality”.tw. |

**Appendix 2: Risk of bias summary table**

|  | | | | | | | | | |
| --- | --- | --- | --- | --- | --- | --- | --- | --- | --- |
| **Author, Year** | **Risk of Bias** | | | | **Applicability** | | | **Overall** | |
|  | 1. Participants | 2. Predictors | 3. Outcome | 4. Analysis | 1. Participants | 2. Predictors | 3. Outcome | **Risk of Bias** | **Applicability** |
| Esteban-Burgos, 2023 | - | + | + | - | - | + | + | - | - |
| Hicks, 2010 | - | + | + | - | - | + | + | - | - |
| Hirdes, 2014 | - | + | + | - | - | + | + | - | - |
| Marsh, 2000 | - | + | + | - | - | + | + | - | - |
| McCusker, 2014 | - | + | + | - | - | + | + | - | - |
| Mitchell, 2004 | + | + | + | + | + | + | + | + | + |
| Mitchell, 2010a | + | + | + | + | + | + | + | + | + |
| Mitchell, 2010b | - | + | + | + | - | + | + | - | - |
| Van der Steen, 2007 | + | + | + | + | + | + | + | + | + |
| Volicer, 1993 | - | + | + | - | - | + | + | - | - |
|  |  |  |  |  |  |  |  |  |  |
|  |  |  |  |  |  |  |  |  |  |

**Appendix 3: Risk of bias and applicability summary**

**
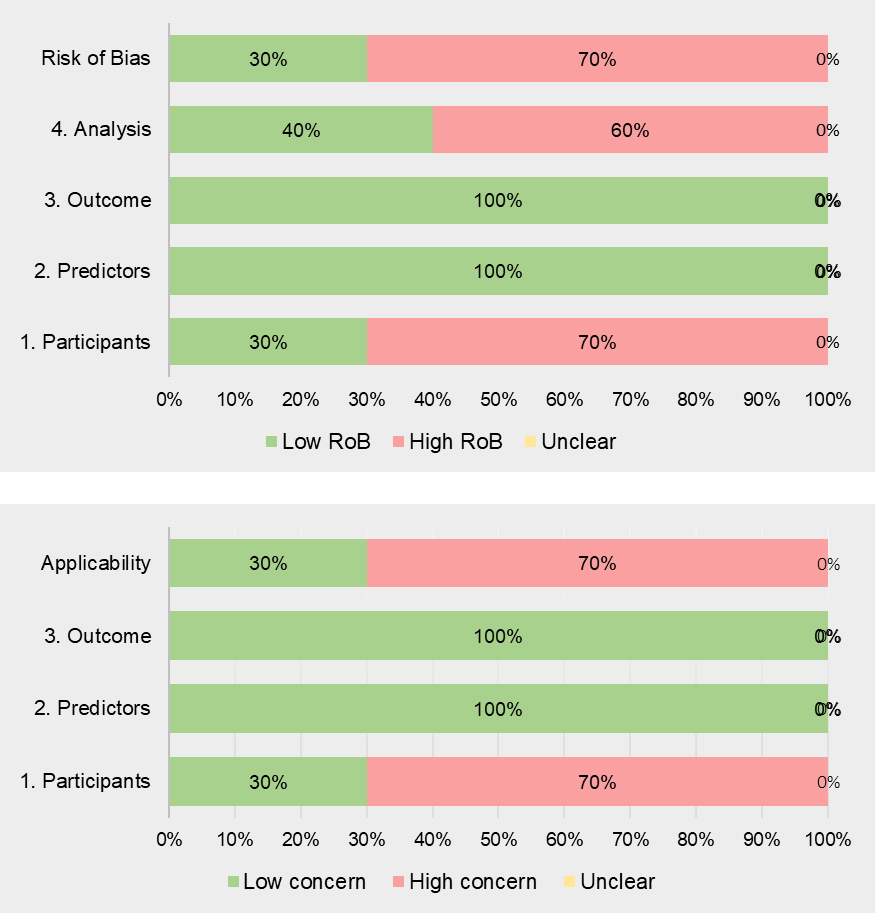
**

| \|  \| \| --- \| |  |  |  |  |  |  |
| --- | --- | --- | --- | --- | --- | --- | --- |
|  |  |  |  |  |  |  |

**Appendix 4: Summary of discrimination and calibration assessment across included studies**

|  | **Number of studies (%)** |
| --- | --- |
| **Assessment of discrimination**  Area Under Curve  Not reported | 6 (46.2)  7 (53.8) |
| **Rating of discrimination**  Outstanding *(≥0.90)*  Excellent *(0.80-0.89)*  Acceptable *(0.70-0.79)*  Poor *(0.50-0.69)*  Discrimination not reported | 0  0  2 (15.4)  4 (30.8)  7 (53.8) |
| **Assessment of calibration**  Calibration plot  Hosmer-Lemeshow test results  Other  Not reported | 1 (7.7)  2 (15.4)  1 (7.7)  9 (69.2) |
| **Rating of calibration by study authors**  Adequate  Poor  No rating provided  Calibration not reported | 3 (23.1)  0  1 (7.7)  9 (69.2) |
